# Volume Fifteen

**Published:** 1875-08

**Authors:** 


### Editorial.

### Volume Fifteen.

With this number we commence the fifteenth volume of the BUFFALO MEDICAL AND SURGICAL JOURNAL, and we have again to thank our friends for the generous support which their valuable contributions have given us. It has been our aim to make the contents of the JOURNAL as full of practical interest as possible, and with this view we have used as much care as possible, both in the original department and in our selections from exchanges. In the coming volume we shall endeavor, under the head of *Medical Notes*, to present our readers with a resumé of the recent literature in the various departments of the medical sciences. These notes will be arranged under the various heads of Anatomy and Physiology, Therapeutics, Surgery, Ophthalmology and Gynæcology, and will be under the supervision of different writers.

Dr. W. W. Miner has kindly consented to assume the department of Surgery, that of Obstetrics and Gynæcology will be conducted by the Junior Editor. Of the other departments we can not yet speak positively, but they will be filled in due time. We ask that our friends, both in the city and country, will send us contributions to our original department, they are daily making observations which should be recorded for the benefit of their fellow practitioners.

